# The Phosphate Inhibition Paradigm: Host and Fungal Genotypes Determine Arbuscular Mycorrhizal Fungal Colonization and Responsiveness to Inoculation in Cassava With Increasing Phosphorus Supply

**DOI:** 10.3389/fpls.2021.693037

**Published:** 2021-06-22

**Authors:** Ricardo Alexander Peña Venegas, Soon-Jae Lee, Moses Thuita, Deusdedit Peter Mlay, Cargele Masso, Bernard Vanlauwe, Alia Rodriguez, Ian R. Sanders

**Affiliations:** ^1^Department of Ecology and Evolution, University of Lausanne, Lausanne, Switzerland; ^2^International Institute for Tropical Agriculture (IITA) Kenya, Nairobi, Kenya; ^3^Tanzania Agricultural Research Institute, Mwanza, Tanzania; ^4^International Institute for Tropical Agriculture (IITA) Cameroon, Yaoundé, Cameroon; ^5^Department of Biology, National University of Colombia, Bogotá, Colombia

**Keywords:** *Rhizophagus irregularis*, intraspecific variation, *Manihot esculenta* Cranz., phosphorus fertilization, phosphate, mycorrhizal symbiosis, inoculation responsiveness, phosphate inhibition

## Abstract

A vast majority of terrestrial plants are dependent on arbuscular mycorrhizal fungi (AMF) for their nutrient acquisition. AMF act as an extension of the root system helping phosphate uptake. In agriculture, harnessing the symbiosis can potentially increase plant growth. Application of the AMF *Rhizophagus irregularis* has been demonstrated to increase the yields of various crops. However, there is a paradigm that AMF colonization of roots, as well as the plant benefits afforded by inoculation with AMF, decreases with increasing phosphorus (P) supply in the soil. The paradigm suggests that when fertilized with sufficient P, inoculation of crops would not be beneficial. However, the majority of experiments demonstrating the paradigm were conducted in sterile conditions without a background AMF or soil microbial community. Interestingly, intraspecific variation in *R. irregularis* can greatly alter the yield of cassava even at a full application of the recommended P dose. Cassava is a globally important crop, feeding 800 million people worldwide, and a crop that is highly dependent on AMF for P uptake. In this study, field trials were conducted at three locations in Kenya and Tanzania using different AMF and cassava varieties under different P fertilization levels to test if the paradigm occurs in tropical field conditions. We found that AMF colonization and inoculation responsiveness of cassava does not always decrease with an increased P supply as expected by the paradigm. The obtained results demonstrate that maximizing the inoculation responsiveness of cassava is not necessarily only in conditions of low P availability, but that this is dependent on cassava and fungal genotypes. Thus, the modeling of plant symbiosis with AMF under different P levels in nature should be considered with caution.

## Introduction

Phosphorus (P) is an essential nutrient for plants that affect the productivity of crops (Karandashov and Bucher, 2005). Unfortunately, P cannot diffuse rapidly in the soil and depletion zones form around root hairs (Schachtman et al., 1998; Vance, 2001). Thus, many plants have difficulty obtaining enough P. Worldwide, 67% of the total farmed land is considered P deficient (Cakmak, 2002). While P limits plant growth in soils globally, this is especially true in the tropics, due to the additional problem of P fixation by aluminum and iron oxides common in tropical acidic soils (Rahman et al., 2018). Consequently, P fertilization is essential in agriculture to support high crop yields.

In nature, plants interact with diverse microorganisms to improve P uptake (Sharma et al., 2013). One of the most important and ubiquitous interactions is the mutualism between plants and arbuscular mycorrhizal fungi (AMF), which occurs in 72% of land plant species (Brundrett and Tedersoo, 2018). In AM symbiosis, the plant provides AMF with sugars and lipids. After the colonization of the roots, the fungus forms an extra-radical mycelial network that extends beyond the P depletion zone and exploits a greater volume of soil than that possible by roots alone (Jansa et al., 2011; Smith and Smith, 2011). The fungi can efficiently take up P from the soil and transfer it to the plant, thus increasing plant productivity (Smith and Read, 2008). The symbiosis occurs in almost all economically important crops worldwide. Because of the capacity of AMF to improve plant P acquisition, there is much interest in applying AMF in agriculture to increase crop yields while simultaneously reducing the amount of P fertilizer applied to crops. This is especially so in the tropics because of the severe difficulties for crops to obtain enough P in much of the agricultural soils.

Based on the results of many experimental studies in the laboratory and greenhouse, it is widely accepted that the development of AMF inside plant roots is regulated by P availability to the plant. *In vitro* experiments have demonstrated that under P limitation, plant roots increase the production of signal molecules stimulating the establishment of the symbiosis (Nagahashi et al., 1996; Nagahashi and Douds, 2000). When P is applied to plants, the plant regulates colonization by AMF such that colonization decreases with increasing available P (Mosse, 1973; Thomson et al., 1986; Smith and Read, 2008; Balzergue et al., 2010; Breuillin et al., 2010). The molecular basis in plants for this P-induced suppression of AMF colonization has also been experimentally demonstrated (Breuillin et al., 2010; Salvioli di Fossalunga and Novero, 2019) as well as how the supply of inorganic P influences the expression of genes regulating P homeostasis in the fungus itself (Ezawa and Saito, 2018). Additionally, studies have demonstrated that applying more P reduces the benefit to plant growth afforded by inoculating the plants with AMF (Bruce et al., 1994; Valentine et al., 2001; Xu et al., 2014). The ability of plants to limit AMF colonization under high P supply is considered a strategy to limit carbohydrate consumption by AMF when the fungi are less needed for P uptake from soil. The negative relationship between P supply and both reduction in colonization and plant benefit has mostly been demonstrated in one-to-one models (one plant cultivar inoculated with one AM fungus isolate), but the same relationship has been observed with a very large variety of different plants and different AMF species and isolates (Mosse, 1973; Bruce et al., 1994; Balzergue et al., 2010; Breuillin et al., 2010). The relationship between increasing P application and decreasing AMF colonization and plant responsiveness to inoculation with AMF is so well-demonstrated experimentally that we refer to it here as the P inhibition paradigm.

Because of the P inhibition paradigm, there is an overriding assumption that if the crop is sufficiently well-fertilized with P and other nutrients, then it will not benefit from inoculation with AMF. However, the markedly phytocentric view in most experimental studies regarding this topic is possibly a serious oversimplification (Smith and Read, 2008) and possibly does not hold true in field situations. Under field conditions, responses to increasing P supply are potentially more complex. Several field trials have reported decreases in AMF colonization of roots and/or mycorrhizal benefit in crops with P fertilization (Thingstrup et al., 1998; Kahiluoto et al., 2001; Al-Karaki, 2006; Verbruggen et al., 2012). However, very low P availability (such as the levels common in some tropical soils) can inhibit AMF colonization so that additions of P are required to stimulate colonization (Bolan et al., 1984; de Miranda and Harris, 1994). More recently, Higo et al. (2020) reported that AMF colonization of tomato roots does not always decrease with increasing P supply in the field in temperate soil, although in a short-term (7 weeks) experiment. At present, the current P inhibition paradigm is yet to be generalized in the field. Moreover, to the knowledge of the authors, there are no published studies testing the P paradigm in globally important crops in tropical soils.

Cassava (*Manihot esculenta* Crantz.) is known to be one of the crops that are most responsive to AMF inoculation (Howeler et al., 1982). This tropical root crop is an important source of carbohydrates for almost 800 million people living in tropical and sub-tropical regions of the world (El-Sharkawy, 2006; FAO, 2013). P availability is a crucial factor determining cassava productivity (Howeler et al., 1982; Smith et al., 2011), and P fertilization is considered essential to improving cassava yields. Pioneering field experiments in Colombia showed that cassava is strongly influenced by AMF inoculation for growth and nutrition in low-P soils (Howeler, 1981; Howeler et al., 1982; Howeler and Sieverding, 1983; Sieverding and Howeler, 1985). The AMF species *Rhizophagus irregularis* is a promising candidate for the inoculation of cassava because of its easy cultivation *in vitro* in a sterile medium and its very wide niche, occurring in agricultural soils on several continents (Ceballos et al., 2013; Savary et al., 2018). The application of *in vitro*-produced *R. irregularis* was shown to improve the productivity of cassava (Ceballos et al., 2013; Aliyu et al., 2019).

Field trials in Kenya and Tanzania have demonstrated that inoculation with different *R. irregularis* isolates, or offspring cultures of those isolates, influenced cassava root productivity enormously by up to a factor of 300% (Ceballos et al., 2019). Remarkably, the plants in those experiments were all fertilized with what is considered an optimal amount of nitrogen (N), P, and potassium (K). These results are inconsistent with the P inhibition paradigm where the supply of sufficient P to cassava would have inhibited AMF colonization and reduced the effects of AMF inoculation on cassava growth to a negligible level. In those experiments, large differences in cassava responsiveness to AMF inoculation were observed among locations and cassava varieties at optimal fertilization levels.

In the present study, we investigated whether the application of different amounts of P fertilizer altered the responsiveness of cassava to inoculation by different *in vitro*-produced *R. irregularis* isolates and their offspring and influenced the colonization of cassava roots by AMF. We conducted field trials at three locations (one in Kenya and two in Tanzania), using a landrace and an improved cassava variety at each location. The soils at the three locations were considered very-low to medium-low P content soils (according to threshold values for cassava growth reported by Howeler, 2012). We hypothesized that (a) following the P inhibition paradigm, the benefit cassava receives from inoculation with AMF decreases with increasing P supply and that AMF colonization will also decrease irrespective of the identity of the AMF identity or cassava variety; (b) genetically different AMF isolates will not have the same effect among cassava varieties and locations under different P fertilization levels. To verify the hypotheses, we performed an experiment at three locations in Africa (Supplementary Figure S1), where a cassava growth response to P fertilization was expected due to the defined low P content of the soils.

## Materials and Methods

### Field Sites

The experiments were established at three different locations: (1) Ukwala-Kawayo, Siaya County, Kenya (00° 15' 12.1" N; 34° 10' 32.7" E); (2) Kayenze, Biharamulo district, Tanzania (03° 12' 3.33" S; 31° 26' 37.18" E); and (3) Kijuka, Sengerema district, Tanzania (02° 35' 20.41" S; 32°35'42.79" E; Supplementary Figure S1). Climatic and soil physical and chemical characteristics of the three locations are shown in Supplementary Tables S1, S2. The soil P content at each of the three locations in this study spanned the range from very low to medium-low according to the specific cassava threshold values (low: 2–4 mg.kg^−1^ and medium: 4–15 mg.kg^−1^) reported by Howeler (2012).

### Plant and Fungal Material

At each location, we planted improved cassava (*M. esculenta*) variety that had been bred for disease resistance and a locally grown landrace (Supplementary Table S3). The choice of which improved variety was planted at each location was based on recommendations by the International Institute of Tropical Agriculture (IITA). The choice of landrace at each location was based on which variety the local farmers frequently cultivated, and the landrace was different at each location. Inoculum of the AMF species *R. irregularis* was produced in an *in vitro* culture system by Symbiom s.r.o. (Lanskroun, Czech Republic) and mixed with a sterile inert carrier (calcified diatomite). The culture conditions, including the type of medium and temperature for incubation, were previously described by Rosikiewicz et al. (2017). Six *R. irregularis* cultures were chosen based on preliminary results of a previous field trial conducted at Ukwala-Kawayo, Kenya that was performed with a greater number of fungal cultures and the cassava landrace Fumba chai (Ceballos et al., 2019). The three *R. irregularis* cultures that resulted in the greatest cassava productivity in that experiment, namely C3.14, C3.16, and C3.22, and the three cultures that gave the lowest cassava productivity, namely C2, C3, and A5.8, were chosen for this study. *Rhizophagus irregularis* C2 and C3 are isolates that originated from the field at Hausweid, Tänikon, Switzerland (Koch et al., 2004). C3.14, C3.16, and C3.22 are progeny cultures of parental isolate C3, each initiated from a single spore of the parental isolate (Ceballos et al., 2019). A5.8 is a progeny culture of parental isolate A5 that was isolated at the same time and from the same field as C2 and C3. We refer to these as progeny or single spore lines (SSLs). The parental isolates have been maintained as *in vitro* cultures in identical conditions at the University of Lausanne since 2000, and the SSL cultures were established *in vitro* in 2015 in the same conditions.

### Design and Establishment of the Field Experiments

Cassava was planted and inoculated in a randomized block design with eight blocks at each location. The experiment comprised eight inoculation treatments with the six *R. irregularis* cultures, as well as two control treatments: no inoculation and inoculation with the carrier but with no fungus. There were two cassava varieties, namely one landrace and one improved variety, and three locations. There were three levels of P fertilization, namely 0, 50, and 100%, where 100% represented the recommended dose for optimal cassava growth (Supplementary Note S1), based on soil chemical analyses at each location. Thus, there were 48 treatment combinations and each of the eight blocks contained all the 48 treatment combinations (Supplementary Figure S2). Plots containing nine plants represented one experimental unit (or replicate) that received one of the 48 treatment combinations, and each plot was surrounded by 16 non-inoculated plants (Supplementary Figure S2). The experiment at each location covered an approximate area of 1 ha with a planting density of 10,000 plants ha^−1^.

Cassava stem cuttings (30 cm long) were inoculated with 1 g of diatomite carrier containing 1,000 fungal spores of a given fungal parental isolate or SSL, and this was placed around the stem of the cassava at planting. Fertilizer was applied between 30 and 45 days after planting (DAP). The amount of fertilizer applied was determined by the initial soil nutrient content, nutritional requirements of cassava, and fertilizer efficiency (Supplementary Note S1). The nutritional requirements, proposed by the IITA, were as follows: 150 kg ha^−1^ N, 40 kg ha^−1^ P, and 180 kg ha^−1^ K (Ezui et al., 2016). The sources of fertilizer applied were urea, triple super phosphate (TSP), and muriate of potash. Cassava plants at different locations received the appropriate amount of P so that at each location, plants in the 100% P treatment were exposed to 40 kg ha^−1^ available P. This was calculated separately for each site using the values of available P obtained in the soil analyses (Supplementary Table S2). The amount of P fertilizer added to the different treatments was adjusted by the dosage of TSP.

In Kayenze and Kijuka (Tz), the trials were established in January 2018 and harvested a year later. In Ukwala-Kawayo (Ke), the trial was sown in March 2018 and also harvested a year later.

### Plant and Fungal Growth Measurements

The colonization of AMF in roots (% colonized root length) and root fresh weight (kg plant^−1^) was measured at harvest time. Cassava fine roots were collected at a depth of 0–30 cm. Plants were uprooted by removing the soil around the stem and starchy roots with a shovel, and then manually pulling them out to assure that many of the fine roots came out with the main starchy root. Then, 3 g of fine roots were collected from the main roots after pulling out the plant from each cassava plant and stained following the method of Koske and Gemma (1989), except trypan blue was replaced by acid fuchsin 0.01%, and checked for AMF colonization using the grid-line intersection method (Giovanetti and Mosse, 1980). Previous studies have shown that fresh and dry root weights are the best traits explaining the cassava responsiveness to inoculation with AMF (Ceballos et al., 2013, 2019). In this study, root fresh weight (kg plant^−1^) showed a strong linear correlation (*R*^2^ > 0.83, *p* < 0.001) with root dry weight (kg plant^−1^) regardless of the cassava variety (Supplementary Figure S3). Therefore, here, we only report further analyses concerning root fresh weight at harvest as it is a good estimator of root biomass production. In addition, we only report on this variable because fresh cassava roots are the edible part of the plant, which directly corresponds to the yield of the crop. Additionally, to have a standardized measure of mycorrhizal effects on plants inoculated with different inoculation treatments, we calculated the direction of the productivity response of plants to inoculation with a given *R. irregularis* culture compared to both non-inoculated plants and plants inoculated with the carrier but without fungus by a modification of mycorrhizal responsiveness (measured as the biomass difference between inoculated plants and non-inoculated controls; Janos, 2007). In this study, we refer to this as inoculation responsiveness. We only discuss the results of inoculation responsiveness where it was observed that both calculations of responsiveness, using either the non-inoculated plants or the carrier without fungus, gave the same result.

### Statistical Analyses

Statistical analyses were conducted with R (R Development Core Team, 2019; v3.5.3) and JMP® v14.2.0 (SAS Institute Inc.). A linear correlation analysis was performed on fresh and dry root weight. Before ANOVA, the data were tested for normality using a Shapiro–Wilk test and for homoscedasticity using Bartlett’s and Levene’s tests. Root fresh weight and AMF colonization (%) were normally distributed but with unequal variance, thus violating the assumptions of conventional ANOVA. Therefore, Welch’s ANOVA was performed, followed by a Games-Howell *post hoc* test.

In this study, there were four main factors: location, cassava variety, P fertilization level, and AMF inoculum. However, because different cassava varieties were planted among locations, and locations differ in soil characteristics (Supplementary Tables S1, S2, S3), the data revealed a significant interaction effect between the two main factors: location and cassava variety. The nature of the data greatly hindered the statistical power of a generalized model-based analysis even with using random factor assumption. Therefore, following the testing of the P fertilization effect on root fresh weight and mycorrhizal colonization rate in a global analysis, we divided the data by location and by cassava variety for further testing the effect of P fertilization and AMF inoculation. The first hypothesis we tested was that AMF colonization and the benefit cassava receive from AMF inoculation decreases with increasing P supply. For this, we first tested if there is any difference in cassava root fresh weight or AMF colonization in both cases of with and without inoculation and by the level of P fertilization in the global dataset. After this, we divided the dataset by location and cassava variety to test the same question. We further calculated the inoculation responsiveness and tested if this was affected by P fertilization in each cassava variety and at each location separately. There were two different inoculation responsiveness measurements calculated using two different controls: non-inoculated plants and plants inoculated with the carrier but without fungus. Therefore, statistical tests were conducted independently for each of the two different calculations of inoculation responsiveness. The second hypothesis was that genetically different AMF isolates will not induce the same inoculation responsiveness among cassava varieties and locations under different P fertilization levels. To test this hypothesis, we compared the inoculation responsiveness of different AMF isolates at each level of P, with each cassava variety, and at each location.

## Results

### Effects of Location, Cassava Variety, and P Fertilization on Root Fresh Weight and AMF Colonization

**Figure 1:**
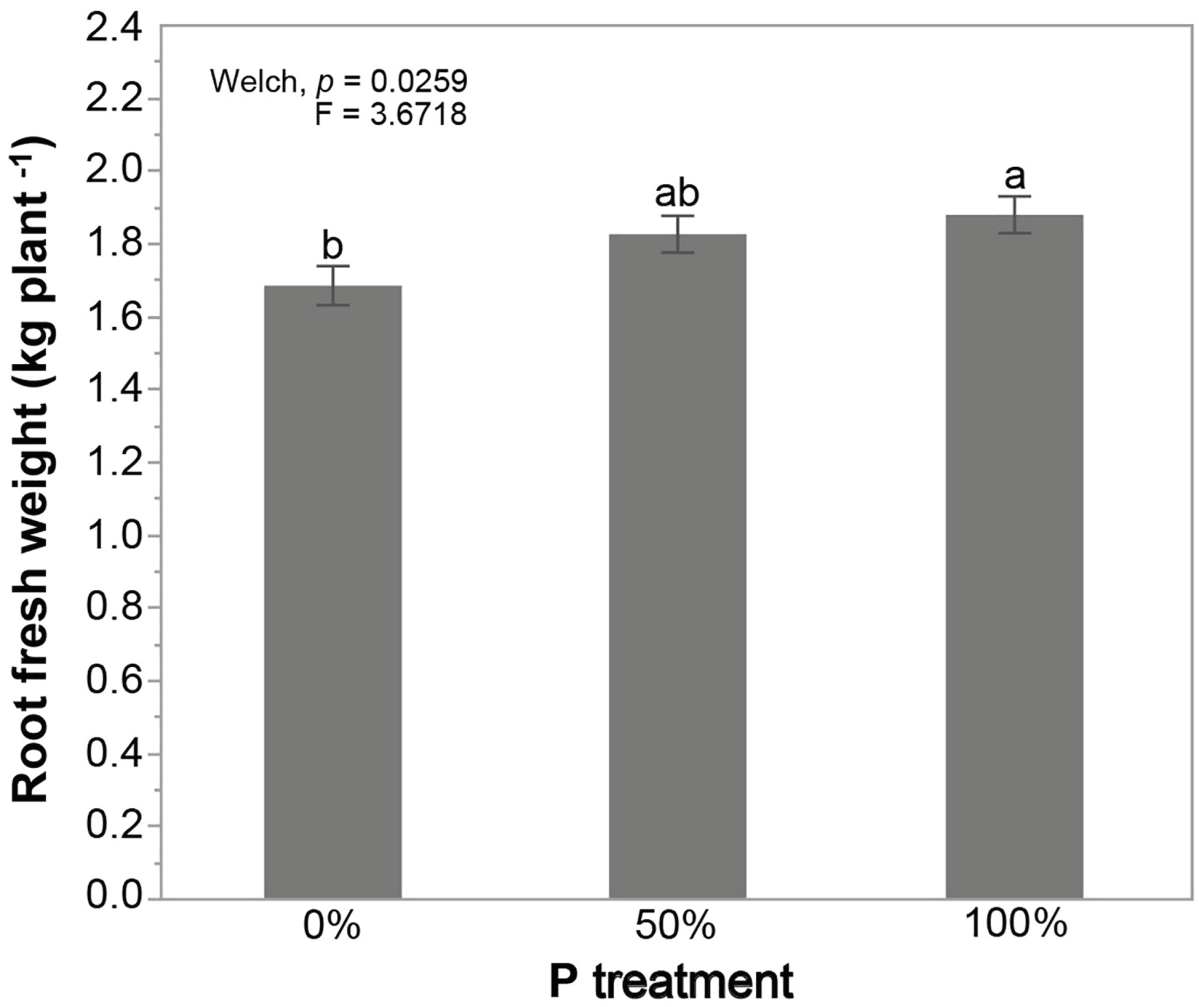
Effects of phosphorus (P) fertilization on cassava root fresh weight averaged over the three locations, all cassava varieties, and all arbuscular mycorrhizal fungi (AMF) treatments. Error bars represent ±SE. Means with different letters are significantly different at *p* < 0.05 according to a Games-Howell *post hoc* test.

We first analyzed the effects of the main factors on cassava root fresh weight or AMF colonization in both cases, with and without inoculation. A global analysis of the whole dataset (including all the data from the three locations, all cassava varieties, and AMF treatments) was conducted first to assess the general effect of each of the main factors on root fresh weight (Supplementary Table S4). The results showed that cassava root fresh weight significantly differed among locations, cassava variety, and P treatment. Although cassava root fresh weight in the whole dataset was significantly greater when plants were fertilized with the 100% P compared to the 0% P treatment, the differences in root weight were extremely small (Figure 1; Supplementary Table S4). There was no significant difference in root weight between the 50 and 100% P treatments. We also performed the same analysis on AMF colonization to see if there were differences among P treatments. However, even though, a mean colonization of over 30% was observed in all P treatments [0% P = 32.93 (mean) ± 1.71 (SE), 50% P = 35.50 ± 1.67, and 100% P = 32.18 ± 1.54], there were no significant differences among the treatments.

**Figure 2:**
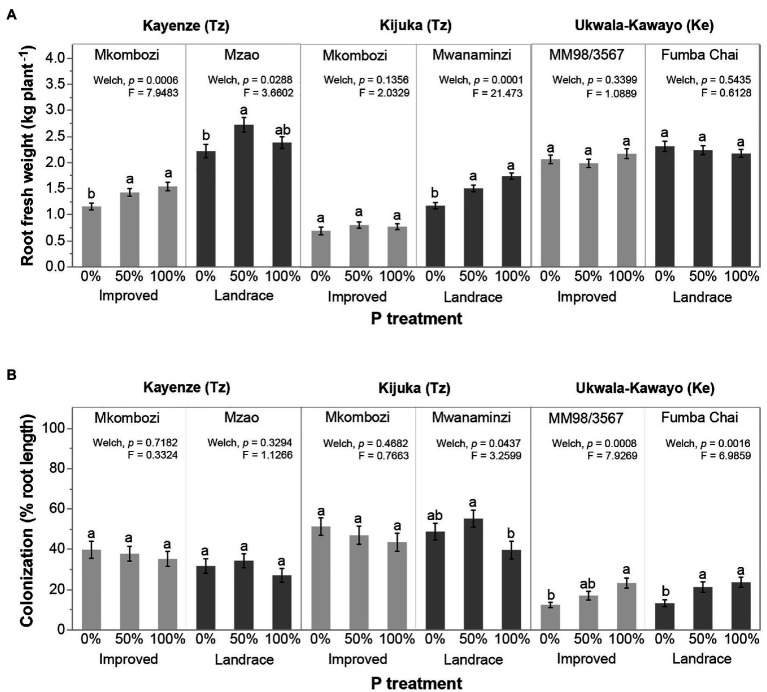
Effects of P fertilization on **(A)** cassava root fresh weight (kg plant^−1^) and **(B)** AMF colonization (% root length) of AMF inoculated treatments at each of the three locations and in the two cassava varieties grown at each location. Error bars represent ±SE. Means with different letters are significantly different at *p* < 0.05 according to a Games-Howell *post hoc* test. The statistical test represents a comparison among P treatments within each cassava variety at each location. Tz, Tanzania; Ke, Kenya.

Because there were large variations in cassava root weight among locations and cassava varieties, and because the cassava varieties were not always the same at each location, an overall analysis considering locations and cassava varieties as random effects resulted in the loss of statistical power. Therefore, we separated the dataset by location and cassava variety to further investigate the effect of P treatment on cassava root fresh weight and AMF colonization. This analysis revealed location-specific and variety-specific responses to P fertilization, which were masked in the analysis of the whole dataset (Figure 2). P fertilization significantly increased root fresh weight in both cassava varieties in Kayenze (Figure 2A). Root fresh weight of the landrace Mwanaminzi variety significantly increased with increasing P fertilization in Kijuka but the improved variety (Mkombozi) was unresponsive (Figure 2A). There was no effect of P fertilization on root fresh weight in Ukwala-Kawayo in either variety.

The fertilization of cassava with different levels of P did not affect the AMF colonization of cassava roots in Kayenze in either variety (Figure 2B). In Kijuka, the colonization of cassava roots by AMF in the improved variety (Mkombozi) was unaffected by P fertilization. Colonization of the roots by AMF in the landrace Mwanaminzi variety was significantly lower at 100% P compared to 50% P fertilization (Figure 2B). There was a significant effect of P fertilization on the AMF colonization in both cassava varieties in Ukwala-Kawayo, where higher P application resulted in higher AMF colonization.

Even though in this study, we were not able to distinguish root colonization of the inoculated AMF from that of the native AMF community, there was no significant difference in root fresh weight and AMF colonization between the non-inoculated controls and inoculation treatments at different levels of P supply, in each plant variety and at each location. In general, there was no significant difference in root fresh weight or AMF colonization observed in non-inoculated controls following different levels of P application (Supplementary Figure S4). Only one case of an increase in root fresh weight at 50 and 100% P compared to 0% P was observed (Mkombozi variety in Kayenze; Supplementary Figure S4). However, the same result was also observed in plants inoculated with AMF (Figure 2; Supplementary Figure S4). At the same time, the effect of P fertilization on AMF colonization was not significant between cassava varieties at any of the locations (Supplementary Figure S4).

### Effect of P Fertilization on Inoculation Responsiveness of Cassava

**Figure 3:**
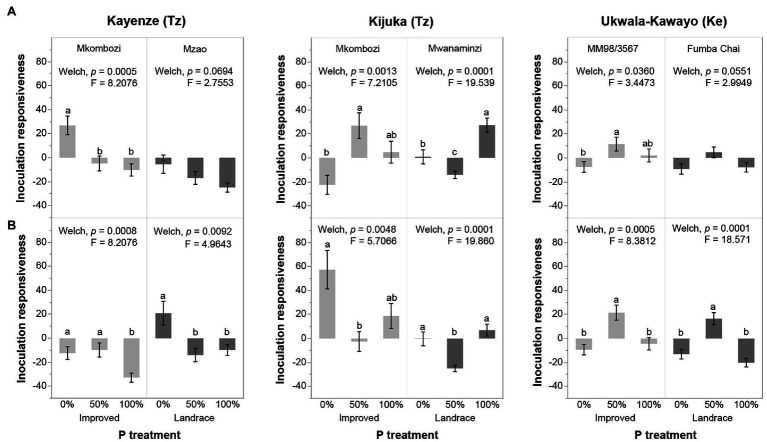
Inoculation responsiveness of cassava to AMF inoculation at the three different P fertilization levels, in two cassava varieties at each of three locations. Inoculation responsiveness was calculated based on cassava root fresh weight response to AMF inoculation compared to **(A)** the non-inoculated treatment and **(B)** the carrier without fungus treatment. Error bars represent ±SE. Means with different letters are significantly different at *p* < 0.05 according to a Games-Howell *post hoc* test. *Post hoc* comparisons were among P treatments within each cassava variety at each location separately. Tz, Tanzania; Ke, Kenya.

To focus on the effect of AMF inoculum, we next tested whether the inoculation responsiveness of cassava decreased with increasing levels of P fertilization. Inoculation responsiveness of cassava was, indeed, significantly affected by the level of P fertilization, but this effect was not the same in each location and each of the two cassava varieties at each location (Figure 3). Inoculation responsiveness decreased with increasing P fertilization in Kayenze, supporting the hypothesis of the P inhibition paradigm (Figures 3A,B). However, different effects of P fertilization on inoculation responsiveness were observed in the other two locations. In Kijuka, the highest inoculation responsiveness was observed in the 100% P fertilization treatment in the cassava landrace Mwanaminzi (Figures 3A,B). In the improved variety Mkombozi, the highest mycorrhizal responsiveness was observed at 50 (Figure 3A) and 0% P (Figure 3B) according to which the control treatment was used to calculate responsiveness to inoculation and did not differ significantly from those in the 100% P treatment. In Ukwala-Kawayo, plants receiving 50% P treatment exhibited greater inoculation responsiveness than plants in the 0 and 100% P treatments (Figures 3A,B).

### Effect of Host and Fungal Identity on Inoculation Responsiveness of Cassava

**Figure 4:**
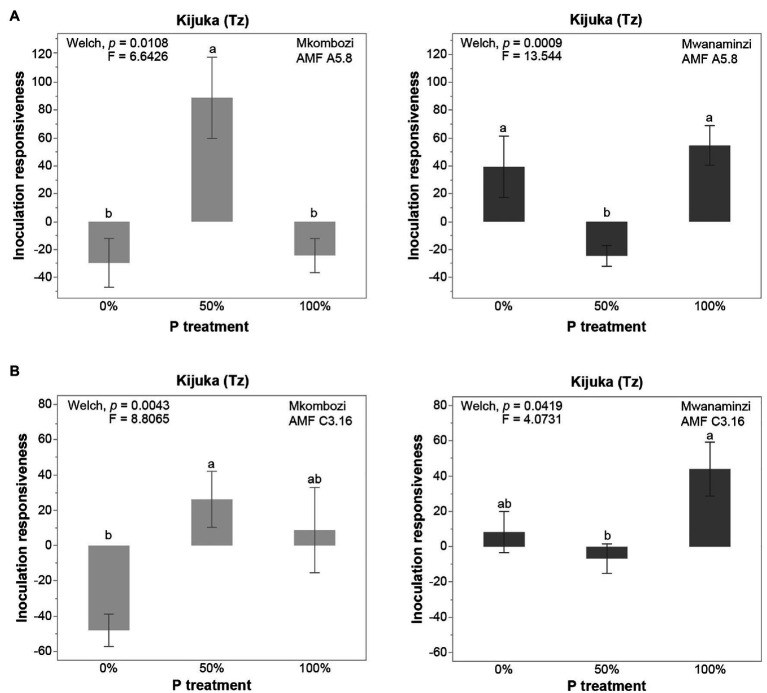
Inoculation responsiveness of Mkombozi and Mwanaminzi varieties to inoculation with *Rhizophagus irregularis* **(A)** culture A5.8 and **(B)** culture C3.16, subjected to different P fertilization treatments at Kijuka. Error bars represent ±SE. Means with different letters are significantly different at *p* < 0.05 according to a Games-Howell *post hoc* test. Tz, Tanzania. Inoculation responsiveness was calculated by comparing inoculated plants to non-inoculated plants.

To investigate the effects of P fertilization on the inoculation responsiveness of cassava to the different AMF inoculation treatments, we further analyzed inoculation responsiveness to P fertilization in each cassava variety and each AMF inoculation treatment. Out of 36 tests, eight tests revealed that inoculation responsiveness of a cassava variety differed among P treatments but depended on the fungus the plants were inoculated with Supplementary Table S5. For example, in Kijuka, the improved cassava variety Mkombozi was significantly more responsive to inoculation with A5.8 at 50% P application, compared to either 0 or 100% P. In the other landrace (Mwanaminzi), the responsiveness to inoculation with A5.8 was significantly lower in the 50% P treatment compared to 0 and 100% P (Figure 4A). At the same location, the responsiveness of the improved variety Mkombozi to inoculation with C3.16 was significantly higher at 50% P than at 0% P. The landrace Mwanaminzi was more responsive to inoculation with C3.16 at 100% P than at 50% P (Figure 4B).

**Figure 5:**
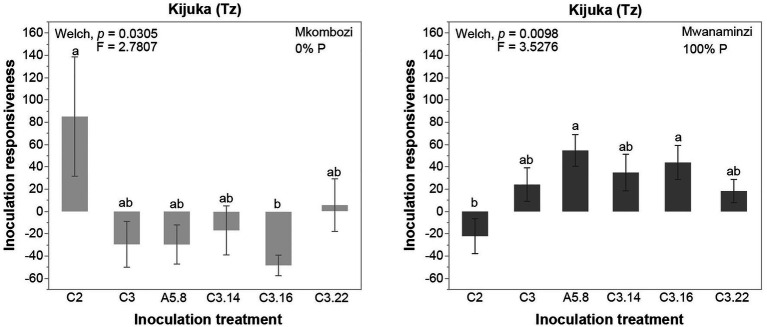
Responsiveness of Mkombozi and Mwanaminzi varieties to inoculation with six *R. irregularis* inoculation treatments and at three levels of P fertilization. Error bars represent ±SE. Means with different letters are significantly different at *p* < 0.05 according to a Games-Howell *post hoc* test. Tz, Tanzania. Inoculation responsiveness was calculated by comparing inoculated plants to non-inoculated plants.

We tested whether the inoculation responsiveness of cassava to different inoculation treatments was the same among cassava varieties and locations under different P fertilization treatments. We observed that inoculation responsiveness to the different AMF treatments, at a given P treatment, was sometimes completely opposing, depending on the cassava variety. For example, the inoculation responsiveness of the Mkombozi variety in Kijuka at 0% P, when inoculated with C2, was positive, while that of plants inoculated with C3.16 showed negative responsiveness (Figure 5). However, the opposite pattern was detected at the same location in the Mwanaminzi variety when plants were fertilized with 100% P (Figure 5). The same pattern of inoculation responsiveness was observed when calculated by comparing inoculated plants to the carrier without fungus treatment (data not shown).

## Discussion

The P inhibition paradigm, where increased P supply suppresses AMF colonization, as well as plant growth responsiveness to the symbiosis, has been fundamental in choosing where the application of AMF is considered appropriate. This study showed that in tropical soils, AMF colonization and responsiveness to inoculation with increasing application of P fertilizer did not follow the trajectory expected from the P inhibition paradigm. Therefore, the first hypothesis of the P inhibition paradigm is rejected. We found that AMF colonization and responsiveness to inoculation, indeed, differ with increasing P application but not in a way that is predicted by the paradigm. Effects of location, cassava variety, and AMF isolate all play a significant role in determining AMF development in cassava roots and plant responsiveness to inoculation at different levels of P application. The results of this study supported the second hypothesis that genetically different AMF isolates do not have the same effect among cassava varieties and locations under different P fertilization levels, even though the AMF isolate identity was not the sole determinant of plant responsiveness to AMF inoculation under different P supply. We discuss these in more detail below and point out where these results help to suggest where research should be focused on to make successful AMF applications more predictive in agriculture.

### Site-Specific Effects

We observed that the way cassava root weight, AMF colonization, and responsiveness to inoculation responded to increasing P application was strongly influenced by location. Despite the soils in these locations being very deficient to moderately deficient in available P, increased P application only induced greater cassava productivity in three of the six cases surveyed in the present study: two varieties at one location, Kayenze, and one variety at Kijuka. However, in these cases, AMF colonization was either unaffected (in Kayenze) or higher in the 50% P treatment than in the 100% P treatment (Kijuka). In Kayenze, responsiveness to inoculation was suppressed with increasing P treatments, but in Kijuka, responsiveness to inoculation was significantly lower in the 50% P treatment compared to both of the other treatments, thus not following the hypothesized pattern. In Ukwala-Kawayo, P application had the complete opposite effect on AMF colonization to that predicted by the P inhibition paradigm, with AMF colonization increasing with increasing P application in both cassava varieties. In these cases, responsiveness to inoculation also did not follow that expected by the P inhibition paradigm.

### Cassava Variety and AMF Identity Effects

Interestingly, the variety of cassava did not play a very important role in determining the AMF colonization responses to increasing P application but greatly affected responsiveness to inoculation. In Kijuka, responsiveness to inoculation in the two varieties was opposite, with responsiveness being highest in one variety at 50% P application and lowest in this treatment in the other variety. Again, these results are not consistent with the P inhibition paradigm. This was especially true for certain fungal treatments where responsiveness to inoculation differed among P treatments but in an opposite way in the two cassava varieties (Figure 5). Responsiveness to inoculation also differed significantly according to AMF identity, but again, which fungus induced the most responsiveness to inoculation differed markedly between no P application and 100% P application. Again, this would not be predicted by the P inhibition paradigm.

### Explanations for the Lack of P-Induced Mycorrhizal Suppression

One possible explanation for the lack of P-induced mycorrhizal suppression is that added P fertilizer was not available to the plants because of P fixation in these soils. Although P availability after the application was not measured, this appears highly unlikely because cassava root weight was P responsive in some cases regardless of location. Furthermore, a significant P application effect on the responsiveness to inoculation, with maximum responsiveness at 50 or 100% P application, is not consistent with the hypothesis that the added P had been fixed in the soil rendering it unavailable. The suppressive effect of P application is known to be attenuated by limited N availability (Nouri et al., 2014), but in these experiments, cassava was supplied with sufficient N.

Among-location differences in P responsiveness may reflect differences in the physical or chemical characteristics of the soils. Such characteristics have previously been shown to affect the AMF-plant symbiosis and alter P uptake and exchange of other nutrients between partners (Liu et al., 2000; Entry et al., 2002; Carrenho et al., 2007; Zaller et al., 2011; Howeler, 2012). However, it cannot explain within-location differences in how cassava varieties or AMF identity influenced plant responsiveness to inoculation at different levels of P fertilization, which were not consistent with that expected by the P inhibition paradigm.

### Arbuscular Mycorrhizal Fungi Community Responses to Changes in P Availability

One large difference between many published experiments that demonstrate the existence of the P inhibition paradigm and these field trials is that most of the studies have been conducted in sterile soil where plants were inoculated with one AMF isolate. In agricultural soils, AMF is almost always present. Thus, many previous studies have assessed how colonization by one AMF taxon, in the absence of other microbes, responds to the effects of P application. In infield trials, AMF colonization represents the response of the pre-existing AMF community, plus the introduced taxa, to different levels of P supply. In several cases, P supply did not suppress mycorrhizal colonization and even had the opposite effect. This indicates that either the taxa we added and/or all or some of the components of the AMF community were not adversely affected by increased P supply. Indeed, the AMF community structure in both the soil and the root can be altered by P supply (Verbruggen et al., 2012; Xu et al., 2018). Thus, we conclude that in the soils where we conducted this study, AMF taxa existed that is not adversely affected by the presence of increased P concentrations.

Because of the pre-existing AMF community and microbiome in agricultural soils, measuring the responsiveness to inoculation in field trials represents the response of plants to the inoculant, in the presence of the AMF community and other components of the microbiome, compared to the effect of the pre-existing AMF community and microbiome alone. This is very different from pot experiments comparing an inoculated plant to a non-mycorrhizal control. In several cases, in this study, we report responsiveness to inoculation that is inconsistent with the P inhibition paradigm. Phosphate availability to roots can be dynamic in time as there should be a gap between the timing of P depletion near AMF hyphae in soil, the growth of new foraging hyphae, and plant uptake of soil P. Consequently, this can affect the temporal patterns of plant P nutrition that can further modulate the AMF community colonizing roots and affect plant responsiveness to inoculation. However, the inoculation responsiveness was measured 1 year after inoculation and is, therefore, based on the accumulation of all possible temporal changes in plant P nutrition and the associated AMF community until the harvest. Therefore, the accumulated effect can help the understanding of the overall impact of inoculation under different P supply. We see the addition of the introduced mycorrhizal taxa as a potential perturbation of the community and that change in the existing microbial community. While we did not measure the effects of inoculation on the AMF community at different levels of P application, it is already known that adding AMF can alter the structure of existing experimental AMF communities (Janoušková et al., 2013). Indeed, adding different *R. irregularis* isolates or their offspring to cassava in tropical soils has been shown to alter both the alpha and beta diversity of AMF communities (Ordoñez et al., 2020). Therefore, we think the effects observed in this study are most likely to be due to mediation by the pre-existing AMF community and the microbiome.

### Conclusion: Challenging the P Inhibition Paradigm

Given the overwhelming number of experimental studies, we do not put into question the existence of the P inhibition paradigm and the mechanism afforded by plants to limit AMF colonization. However, the results of this study strongly suggest that the paradigm cannot be used as a valid generalization for whether or not inoculation will be effective in tropical soils or that AMF should only be applied in P-deficient soils in the absence of P fertilization. The fact that the location, plant variety, and AMF identity all play a role in how P fertilization affects responsiveness to inoculation means that we do not currently have a way of predicting where and in what management conditions, AMF inoculation will be effective. Despite this, the fact that inoculation with different *R. irregularis* strains, in the presence of a pre-existing AMF community and with 100% P fertilization, can alter cassava productivity by up to 300% in these soils (Ceballos et al., 2019) shows that finding the predictors of mycorrhizal responsiveness is a highly pertinent and valuable research pursuit. We propose that this could be addressed by measuring responsiveness to inoculation and screening AMF communities in roots in a large-scale set of field trials, replicated across large edaphic gradients and environmental conditions, as well as with different cassava varieties. A metadata set generated from such trials would allow researchers to search for associations among responsiveness to inoculation, the identity of the fungal inoculant, cassava variety, and climatic and edaphic variables, and above all, the composition of the soil microbial community. We propose that this would allow researchers to predict which combination of factors will give optimal responsiveness to AMF inoculation in a given location.

## Data Availability Statement

The original data collected in the present study are included in the article/Supplementary Material. Further inquiries can be directed to the corresponding author.

## Author Contributions

RAPV conceived and conducted the experiments, analyzed the data, interpreted the data, and wrote the manuscript. S-JL analyzed and interpreted the data, and wrote the manuscript. MT conceived and conducted the experiments. DM conducted the experiments in Tanzania. CM and BV conceived the experiments. AR and IS conceived the experiments, interpreted the data, and wrote the manuscript. All authors contributed to the article and approved the submitted version.

### Conflict of Interest

The authors declare that the research was conducted in the absence of any commercial or financial relationships that could be construed as a potential conflict of interest.
