# Supplementary material for: The Phosphate Inhibition Paradigm: Host and Fungal Genotypes Determine Arbuscular Mycorrhizal Fungal Colonization and Responsiveness to Inoculation in Cassava With Increasing Phosphorus Supply
Source: Front Plant Sci. 2021 Jun 22;12:693037. doi: 10.3389/fpls.2021.693037 (PMC8258410; doi:10.3389/fpls.2021.693037)
Supplement: Supplementary file 2 [file Data_Sheet_2.PDF]

The Phosphate Inhibition Paradigm: Host and Fungal Genotype Determine Arbuscular Mycorrhizal Fungal Colonization and Responsiveness to Inoculation in Cassava with Increasing Phosphorus Supply (Peña *et al*)

SUPPLEMENTARY INFORMATION

This document contains supplementary note 1, supplementary tables 1-5 and supplementary figures 1-4.

Note S1. Optimal fertilization and phosphate fertilization for cassava

Finding optimal nutritional levels for plant growth and productivity is crucial in agriculture as it is directly related to the cost of fertilization and its outcome. Multiple studies have revealed that there are optimal levels of nutrients that can be tuned for maximizing the potential yield of a crop. This often depends on the crop variety (FAO, 2006; Osvalde, 2011). Phosphate fertilization is an important factor determining cassava yield. The optimal nutritional requirements of different cassava varieties is poorly studied. There is no specific guideline for each variety, although there is a general published guideline for optimal nutritional requirements of cassava (Howeler, 2012).

In this study, soil in the three locations differed in the amount of initial available P. However, in all three locations, the amount of available P in the soil is considered far lower than the optimal amount according to Howeler (2012). At each location, the 100% P treatment corresponded to the recommended dose (Ezui *et al.,* 2019), which was adjusted according to the initial soil nutrient content at each location. Consequently, in these experiments, the theoretical amount of available P was the same at all locations in the 100% P and 50% P treatments. At 0%P, as no additional P fertilizer was added, P availability differed among locations according to the values shown in Table S2. In all locations, all plants received the optimal recommended amount of N and K according to (Ezui *et al.,* 2019) which was adjusted according to the initial soil nutrient content at each location. Therefore, cassava growth should not have been limited by N or K availability.

The results showed that the general recommended P dose for cassava should be taken with caution as it is not necessarily the optimal dose for the varieties we planted at those locations. At these locations, similar benefits in yield could be achieved by fertilizing with half of the recommended dose (Figure 1). This highlights the importance of on-going projects for constructing better decision tools for cassava nutrition (e.g. http://acai-project.org/).

Table S1. Climatic and soil physical characteristics at the three locations where the experiments were conducted.

Parameter	Location		
	Kayenze (Tanzania)	Kijuka (Tanzania)	Ukwala-Kawayo (Kenya)
Altitude (m.a.s.l)	1225	1200	1250
Latitude	03° 12' 3.33" S	02° 35' 20.41" S	00° 15’ 12.1’’ N
Longitude	31° 26' 37.18" E	32°35'42.79"E	34° 10’ 32.7” E
Annual temperature (°C)	20.6	22	22.3
Relative humidity (%)	78	68	75
Annual precipitation (mm)	987	1059	1468
Cropping history	Cassava	Maize - bean intercropped	Maize - bean intercropped
Soil Texture	Sand	Sandy loam	Clay
Sand (%)	89.04	79.78	12
Silt (%)	6.11	12.11	16
Clay (%)	4.85	8.11	72
AEZ	Ka 2	Me 1	LM 1

m.a.s.l: meters above sea level; AEZ: agro-ecological zones according to Jaetzold et al. (2006) and Masuki et al. (2016).

Table S2. Soil chemical characteristics at a depth of 30 cm at the three locations where the experiments were conducted. The soil analysis was conducted by commercial company (SGS, Kenya; https://www.sgs.co.ke/). The protocol for soil analyses was as follows. Soils were air-dried, ground and passed through a 2 mm sieve. Samples were further ground and passed through a 0.5 mm sieve before C and N analyses. Soil pH was determined in a 1:2.5 (w/v) soil:water suspension. Organic C was determined by chromic acid digestion and spectrophotometric analysis (Heanes, 1984). Total N was determined after wet acid digest (Buondonno et al., 1995) and analyzed by colorimetric analysis (Anderson and Ingram, 1993). Exchangeable cations (Ca, Mg, K and Na) and micro elements (Cu, Zn, Mn, and Fe) were extracted using the Mehlich-3 procedure and determined by flame atomic absorption spectrophotometry. Available P, extracted using the Mehlich-3 procedure, was analysed using the molybdate blue procedure described by Murphy and Riley (1962). Exchangeable acidity was extracted with 1M KCl and quantified by titration.

	**Kayenze (Tanzania)**		**Kijuka (Tanzania)**		**Ukwala-Kawayo (Kenya)**	
**Properties**	**Mean**	**Standard deviation**	**Mean**	**Standard deviation**	**Mean**	**Standard deviation**
pH (Water)	5.68	0.12	6.19	0.03	5.81	0.02
Ca (cmol(+).kg^{-1})	0.31	0.10	0.40	0.07	5.26	0.06
Mg (cmol(+).kg^{-1})	0.25	0.07	0.37	0.05	1.49	0.02
K (cmol(+).kg^{-1})	0.14	0.07	0.16	0.01	0.60	0.01
Exchange Acidity (cmol(+).kg^{-1})	0.07	0.01	0.01	0.01	0.07	0.01
P (mg.kg^{-1})	5.04	0.14	2.53	0.08	1.30	0.03
Zn (mg.kg-^{1})	0.39	0.01	0.35	0.02	5.04	0.02
Cu (mg.kg^{-1})	0.50	0.01	0.64	0.01	8.78	0.08
Mn (mg.kg^{-1})	19.12	3.75	100.97	0.76	415.68	0.77
Fe (mg.kg^{-1})	74.22	2.95	55.45	0.52	67.99	0.85
Org C (%)	0.68	0.02	0.64	0.03	1.98	0.02
Total N (%)	0.04	0.00	0.04	0.00	0.14	0.00
C/N	17.30	0.42	17.35	0.65	14.12	0.16

Table S3. Description of cassava varieties used in the field experiments.

Location	Variety	Origin	Disease resistance (CMD* and CBSD**)
Kayenze, Tanzania	Mzao	Land race, Tanzania	susceptible
	Mkombozi	Improved, International Institute of Tropical Agriculture (IITA)	resistant
Kijuka, Tanzania	Mwanaminzi	Land race, Tanzania	susceptible
	Mkombozi	Improved, International Institute of Tropical Agriculture (IITA)	resistant
Ukwala-Kawayo, Kenya	Fumba Chai	Land race, Uganda	susceptible
	MM98/3567	Improved, International Institute of Tropical Agriculture (IITA)	resistant

*Cassava mosaic virus disease; **Cassava brown streak virus disease

Table S4. Results of Welch's ANOVA on root fresh weight (kg plant $^{-1}$) for each main factor. Means with different letters are significantly different at $p < 0.05$ according to a Games-Howell post-hoc test. Values highlighted in green are plotted in Figure 1.

Factor	Welch's ANOVA					
	F ratio	df1	df2	*p*	Level	Mean (± standard error)
Location	211.5301	2	710.77	<0.0001	Kayenze, Tanzania	2.02 ± 0.06 b
					Kijuka, Tanzania	1.16 ± 0.04 c
					Ukwala-Kawayo, Kenya	2.20 ± 0.04 a
Variety	144.3495	4	483.81	<0.0001	Mkombozi	1.10 ± 0.04 a
					Mzao	2.62 ± 0.09 a
					Mwanaminzi	1.52 ± 0.05 a
					MM98/3567	2.12 ± 0.05 a
					Fumba Chai	2.27 ± 0.05 b
P treatment	3.6718	2	736.17	0.0259	0%	1.69 ± 0.05 b
					50%	1.83 ± 0.05 ab
					100%	1.88 ± 0.05 a
Inoculation treatment	0.4254	7	470.28	0.8865	C2	1.82 ± 0.09
					C3	1.75 ± 0.08
					A5.8	1.80 ± 0.08
					C3.14	1.70 ± 0.08
					C3.16	1.88 ± 0.09
					C3.22	1.79 ± 0.08
					Ca	1.78 ± 0.08
					No	1.83 ± 0.09
Inoculation treatment without controls	0.5455	5	383.91	0.7417	C2	1.82 ± 0.09
					C3	1.75 ± 0.08
					A5.8	1.80 ± 0.08
					C3.14	1.70 ± 0.08
					C3.16	1.88 ± 0.09
					C3.22	1.79 ± 0.08

Table S5a. Results of Welch's ANOVA on inoculation responsiveness to test for a P treatment effect on cassava root weight at each location and with each cassava variety and with each fungal inoculum. Responsiveness was calculated by comparing inoculated to non-inoculated plants. Means with different letters are significantly different at $p < 0.05$ according to a Games-Howell post-hoc test.

Location	Variety	Welch's ANOVA						
		F ratio	df1	df2	*p*	AMF	P treatment	Mean (± standard error)
Kayenze, Tanzania	Mkombozi (improved)	1.1175	2	12.65	0.3573	C2	0%	17.88 ± 20.25
							50%	-10.96 ± 8.11
							100%	-16.76 ± 10.08
		0.4611	2	13.48	0.6402	C3	0%	16.61 ± 16.63
							50%	-0.45 ± 15.46
							100%	-2.54 ± 10.93
		4.4290	2	13.85	0.0326	A5.8	0%	54.04 ± 17.34 a
							50%	4.33 ± 16.36 ab
							100%	-12.65 ± 13.74 b
		2.3311	2	9.09	0.1524	C3.14	0%	97.00 ± 50.53
							50%	-21.11 ± 16.77
							100%	-1.70 ± 21.42
		4.3277	2	13.23	0.0358	C3.16	0%	21.08 ± 10.87 a
							50%	-14.19 ± 14.81 b
							100%	-24.90 ± 11.51 b
		0.1368	2	12.90	0.8734	C3.22	0%	6.59 ± 16.50
							50%	-4.99 ± 14.21
							100%	0.95 ± 9.47
	Mzao (landrace)	2.2681	2	12.76	0.1436	C2	0%	34.84 ± 23.86
							50%	-18.09 ± 13.85
							100%	-20.85 ± 9.70
		2.0089	2	13.61	0.1720	C3	0%	-35.59 ± 12.30
							50%	2.68 ± 15.57
							100%	-30.69 ± 10.08
		0.5344	2	13.11	0.5983	A5.8	0%	-2.38 ± 23.87
							50%	-28.91 ± 12.04
							100%	-17.94 ± 10.47
		0.0564	2	9.06	0.9455	C3.14	0%	-27.66 ± 16.89
							50%	-30.32 ± 8.97
							100%	-26.50 ± 6.33
		0.4753	2	13.24	0.6319	C3.16	0%	0.74 ± 14.06
							50%	-13.69 ± 10.03
							100%	1.99 ± 18.00
		0.1223	2	13.24	0.8859	C3.22	0%	-8.26 ± 10.32
							50%	-16.24 ± 15.78
							100%	-16.00 ± 17.15

Table S5b. Results of Welch's ANOVA on inoculation responsiveness to test for a P treatment effect on cassava root weight at each location and with each cassava variety and with each fungal inoculum. Responsiveness was calculated by comparing inoculated to non-inoculated plants. Means with different letters are significantly different at $p < 0.05$ according to a Games-Howell post-hoc test. Values highlighted in green are plotted in Figure 4.

Location	Variety	Welch's ANOVA						
		F ratio	df1	df2	*p*	AMF	P treatment	Mean (± standard error)
Kijuka, Tanzania	Mkombozi (improved)	3.0231	2	11.76	0.0872	C2	0%	85.15 ± 53.48
							50%	6.28 ± 23.69
							100%	-35.41 ± 13.56
		1.0852	2	13.75	0.3651	C3	0%	-29.48 ± 10.54
							50%	3.97 ± 24.51
							100%	9.93 ± 17.54
		6.6426	2	12.57	0.0108	A5.8	0%	-29.62 ± 17.57 b
							50%	88.95 ± 29.14 a
							100%	-24.36 ± 12.25 b
		0.0404	2	9.19	0.9606	C3.14	0%	-16.67 ± 22.02
							50%	-11.27 ± 11.34
							100%	-16.59 ± 18.63
		8.8065	2	12.16	0.0043	C3.16	0%	-48.05 ± 9.21 b
							50%	26.18 ± 15.89 a
							100%	8.78 ± 24.18 ab
		0.5337	2	13.77	0.5981	C3.22	0%	5.37 ± 23.62
							50%	37.45 ± 31.96
							100%	37.23 ± 23.25
	Mwanaminzi (landrace)	0.1001	2	11.98	0.9055	C2	0%	-11.27 ± 18.24
							50%	-16.40 ± 7.42
							100%	-22.15 ± 15.68
		5.3539	2	13.36	0.0196	C3	0%	18.54 ± 22.16 a
							50%	-35.89 ± 12.17 b
							100%	24.14 ± 15.06 a
		13.5435	2	11.93	0.0009	A5.8	0%	39.40 ± 22.10 a
							50%	-24.57 ± 7.44 b
							100%	54.72 ± 14.20 a
		2.6413	2	9.39	0.1230	C3.14	0%	0.64 ± 7.38
							50%	-8.96 ± 8.44
							100%	34.94 ± 16.42
		4.0731	2	13.21	0.0419	C3.16	0%	8.25 ± 11.67 ab
							50%	-6.80 ± 8.32 b
							100%	43.95 ± 15.29 a
		2.8685	2	13.05	0.0928	C3.22	0%	-13.91 ± 11.38
							50%	-9.44 ± 6.46
							100%	18.31 ± 10.44

Table S5c. Results of Welch's ANOVA on inoculation responsiveness to test for a P treatment effect on cassava root weight at each location and with each cassava variety and with each fungal inoculum. Responsiveness was calculated by comparing inoculated to non-inoculated plants. Means with different letters are significantly different at $p < 0.05$ according to a Games-Howell post-hoc test.

Location	Variety	Welch's ANOVA						
		F ratio	df1	df2	*p*	AMF	P treatment	Mean (± standard error)
Ukwala-Kawayo, Kenya	MM98/3567 (improved)	1.6868	2	13.89	0.2209	C2	0%	-18.06 ± 12.08
							50%	11.92 ± 12.06
							100%	8.39 ± 14.75
		0.3378	2	13.51	0.7192	C3	0%	7.55 ± 13.11
							50%	20.44 ± 20.59
							100%	-2.20 ± 17.31
		0.3648	2	12.82	0.7013	A5.8	0%	-3.62 ± 8.62
							50%	13.94 ± 19.95
							100%	4.13 ± 12.45
		2.9997	2	13.55	0.0835	C3.14	0%	-25.97 ± 8.22
							50%	10.94 ± 12.20
							100%	-14.01 ± 11.31
		0.8339	2	13.42	0.4557	C3.16	0%	-2.55 ± 9.29
							50%	18.93 ± 15.50
							100%	11.67 ± 12.34
		0.0502	2	13.57	0.9512	C3.22	0%	11.60 ± 17.73
							50%	7.22 ± 11.39
							100%	4.19 ± 14.40
	Fumba Chai (landrace)	2.7852	2	13.50	0.0971	C2	0%	-1.85 ± 13.53
							50%	-10.57 ± 8.49
							100%	23.15 ± 11.16
		2.9709	2	13.74	0.0847	C3	0%	4.77 ± 11.09
							50%	14.26 ± 12.45
							100%	-20.76 ± 9.00
		0.6413	2	13.19	0.5423	A5.8	0%	-20.49 ± 10.02
							50%	-9.37 ± 9.50
							100%	-2.87 ± 12.27
		4.1921	2	12.10	0.0414	C3.14	0%	-20.01 ± 9.95 b
							50%	20.48 ± 11.21 a
							100%	-13.30 ± 4.96 ab
		1.5100	2	12.86	0.2576	C3.16	0%	-3.94 ± 7.68
							50%	19.35 ± 10.60
							100%	2.32 ± 18.37
		0.1880	2	10.33	0.8314	C3.22	0%	-6.18 ± 13.19
							50%	-6.56 ± 9.92
							100%	-12.02 ± 3.95

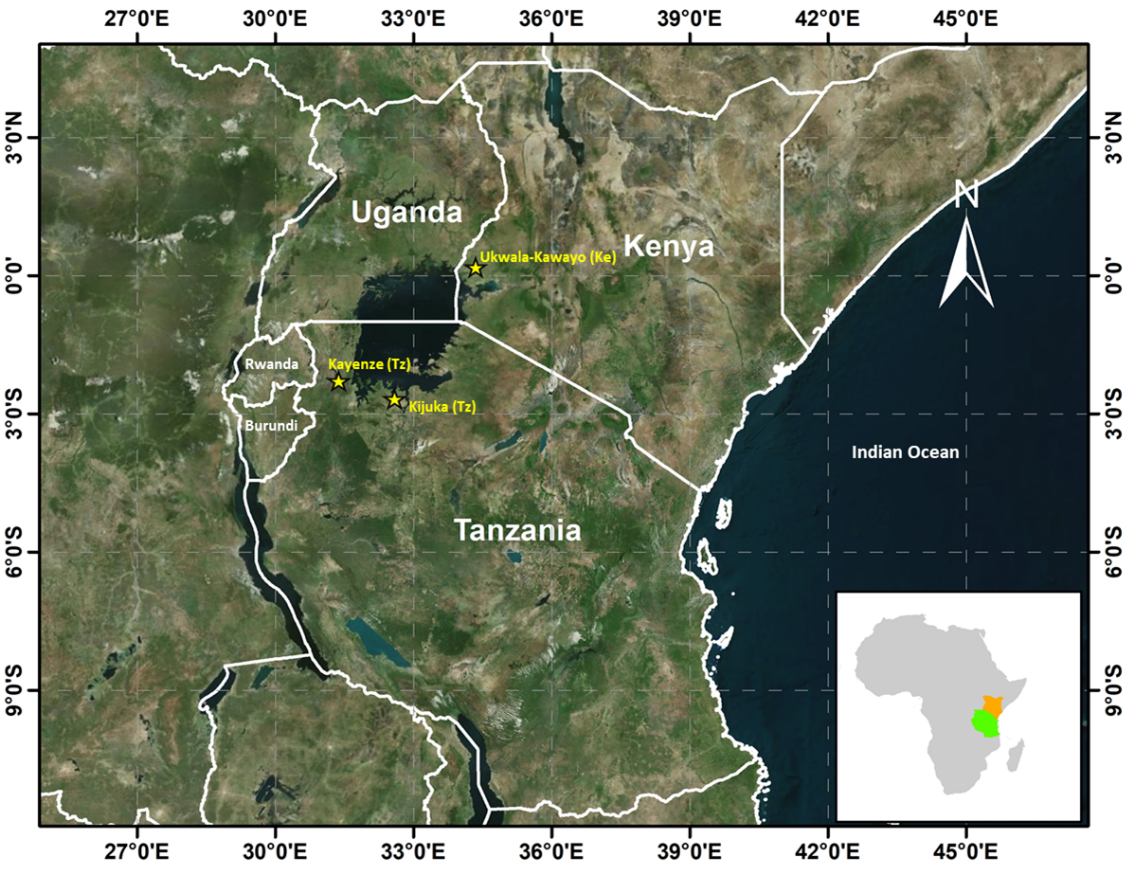

Figure S1. Location of the three locations where the experiments were conducted in Kenya and Tanzania. Map made in ArcGIS 10.4. Base map taken from google maps © and inset map taken from http://www.maplibrary.org/library/stacks/Africa/index.htm, accessed 01/04/2020.

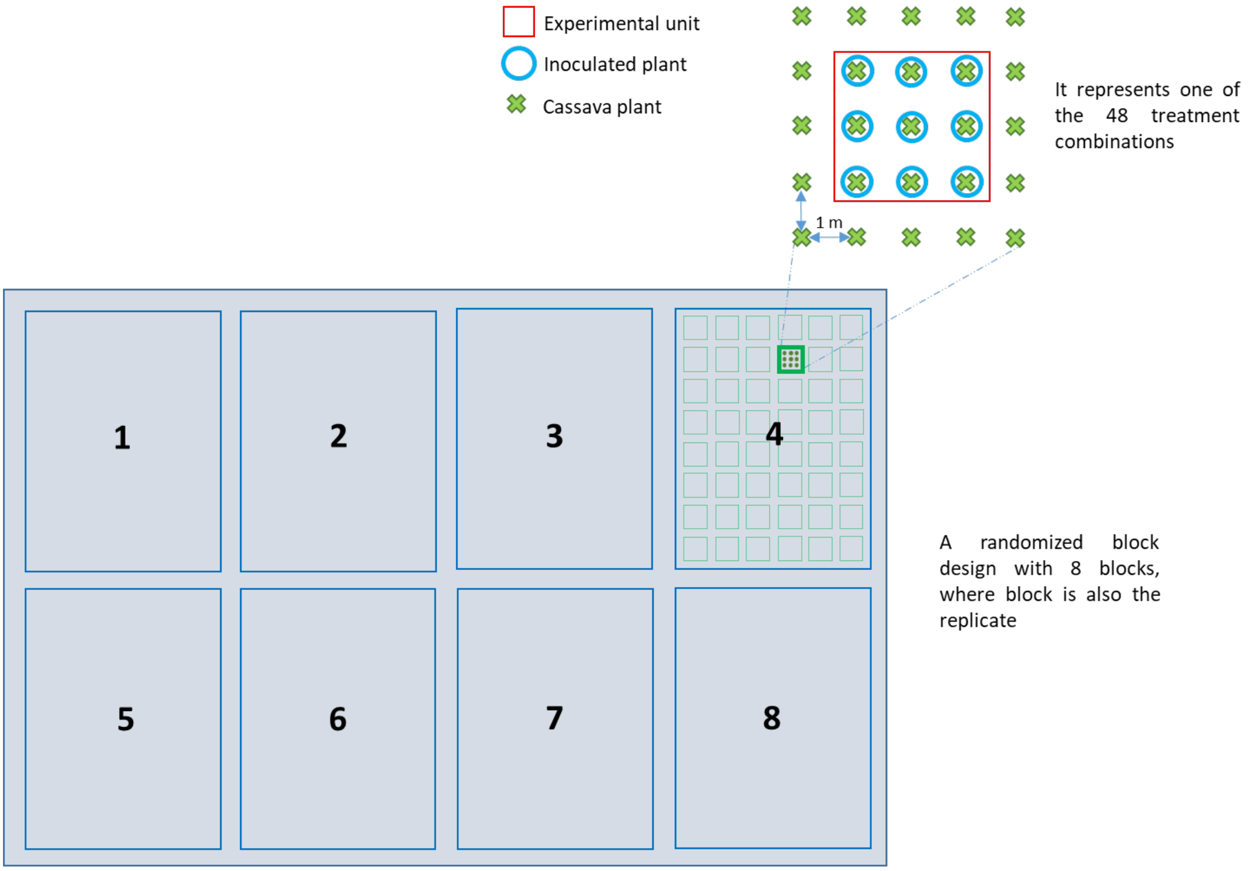

Figure S2. Schematic representation of experimental layout in the field, where a plot of nine plants represented an experimental unit that was subjected to one of the 48 experimental treatment combinations of P fertilizer application, cassava variety and inoculation treatment. There was one replicate of each treatment combination per block and 8 blocks. Treatment positions within the block were randomized among blocks.

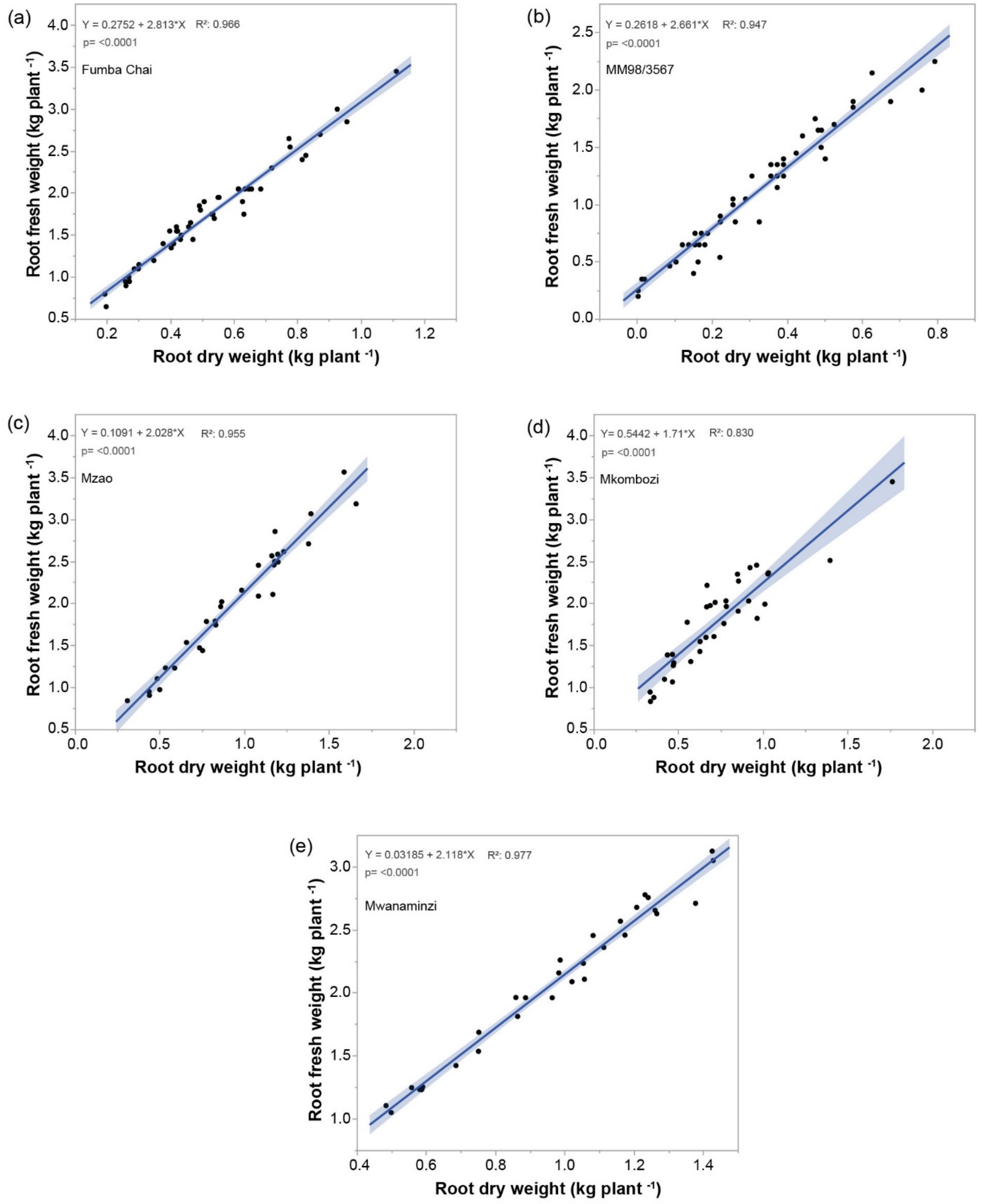

Figure S3. Relationship between fresh root weight and root dry weight per plant in different cassava varieties and different locations. a. Ukwala-Kawayo (Kenya), land race Fumba Chai, b. Ukwala-Kawayo (Kenya), cultivar MM98/3567, c. Kayenze (Tanzania), land race Mzao, d. Kayenze (Tanzania), cultivar Mkombozi, e. Kijuka (Tanzania), land race Mwanaminzi. The regression line is coloured in blue and the light blue shading represents the 95% confidence intervals of the model.

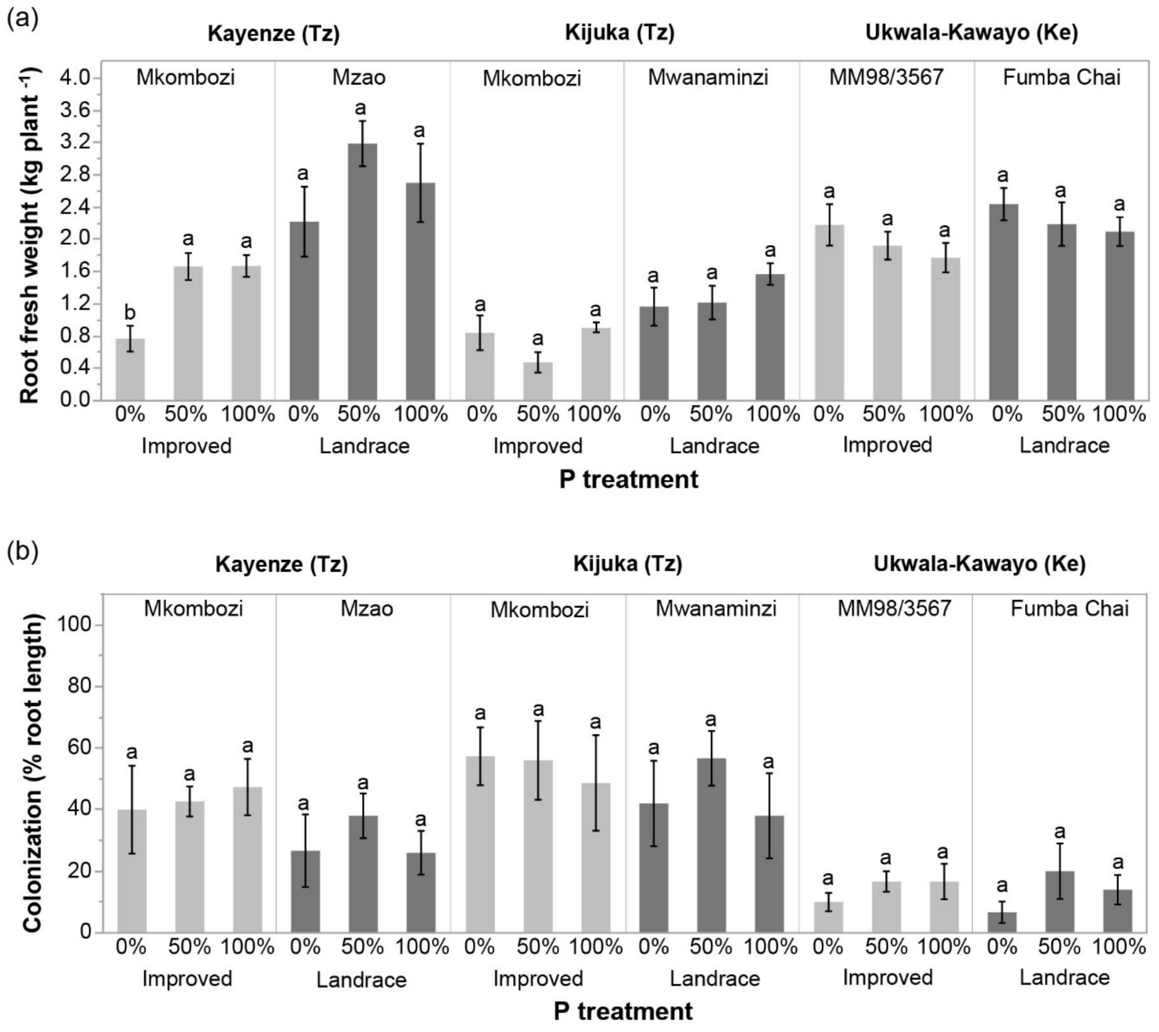

Figure S4. Effects of P fertilization on **(a)** cassava root fresh weight (kg plant $^{-1}$) and **(b)** AMF colonization (% root length) in non-inoculated plants at each of the three locations and the in the two cassava varieties grown at each location. Error bars represent ± S.E. Means with different letters are significantly different at $p < 0.05$ according a Games-Howell post-hoc test. Comparison among P treatments within each cassava variety at each location. Tz: Tanzania and Ke: Kenya.
